# RETIRING AFTER A JOB WELL DONE: HOW SUBJECTIVE CAREER EVALUATIONS IMPACT RETIREMENT ADJUSTMENT

**DOI:** 10.1093/geroni/igad104.3575

**Published:** 2023-12-21

**Authors:** Orlaith Tunney, Kène Henkens, Hanna van Solinge

**Affiliations:** Netherlands Interdisciplinary Demographic Institute-KNAW, The Hague, Zuid-Holland, Netherlands; Netherlands Interdisciplinary Demographic Institute (NIDI), The Hague, Zuid-Holland, Netherlands; Netherlands Interdisciplinary Demographic Institute-KNAW, The Hague, Zuid-Holland, Netherlands

## Abstract

An individual’s past, and how they reflect on it, may influence current and future well-being, with recent qualitative studies suggesting retirees’ reflections on their careers may relate to well-being in retirement. Establishing factors that may promote successful transition to retirement is vital. Particularly in light of population ageing and the subsequent increase in both the number of retirees and the length of retirement. The aim of our study was to investigate the impact subjective career evaluations on retirement adjustment at follow-up. Using data from across three waves of the NIDI Pension Panel Study, (conducted in 2015,2018, and 2023) we examined the pre-retirement career evaluations of a sample of Dutch older workers (N= 3566), factors associated with these evaluations, and the impact of subjective career evaluations on retirement at five or eight-year follow-up. The results of our ordinal logistic regression models showed that how individuals evaluated both the work (b= 0.15 p=.001) and family (b= 0.12 p=.001) domain of career was positively associated with retirement adjustment, with those who evaluated their careers more favorably reporting fewer problems adjusting to retirement. Our results demonstrate the long-term impact of subjective career evaluations and highlight the need for additional research evaluating their predictive utility for other important outcomes in older adulthood such as life satisfaction and health. These findings could also be applied to identify and deliver interventions for those at risk of less favorable retirement outcomes.

